# Prospective association between ultra-processed food consumption and incident depressive symptoms in the French NutriNet-Santé cohort

**DOI:** 10.1186/s12916-019-1312-y

**Published:** 2019-04-15

**Authors:** Moufidath Adjibade, Chantal Julia, Benjamin Allès, Mathilde Touvier, Cédric Lemogne, Bernard Srour, Serge Hercberg, Pilar Galan, Karen E. Assmann, Emmanuelle Kesse-Guyot

**Affiliations:** 1 Equipe de Recherche en Epidémiologie Nutritionnelle (EREN), Université Paris 13, Centre d’Epidémiologie et Statistiques Sorbonne Paris Cité, Inserm (U1153), Inra (U1125), Cnam, COMUE Sorbonne Paris Cité, 74 rue Marcel Cachin, 93017 Bobigny, France; 2 Département de Santé Publique, Hôpital Avicenne, F-93017 Bobigny, France; 3 Faculté de Médecine, Université Paris Descartes, Sorbonne Paris Cité, Paris, France; 4 Service de Psychiatrie et Addictologie de l’adulte et du sujet âgé, AP-HP, Hôpital européen Georges-Pompidou, Paris, France; 5 Institut Psychiatrie et Neurosciences, Inserm (U1266), UMR-S 1266, Paris, France

**Keywords:** Mental health, Depression, Ultra-processed food, Prospective cohort

## Abstract

**Background:**

Ultra-processed food (UPF) consumption has increased over the last decades in Westernized countries. Our objective was to investigate for the first time the association between the proportion of UPF (%UPF) in the diet and incident depressive symptoms in the NutriNet-Santé cohort.

**Methods:**

The sample included 20,380 women and 6350 men (aged 18–86 years) without depressive symptoms at the first Center for Epidemiologic Studies Depression Scale (CES-D) measurement, using validated cut-offs (CES-D score ≥ 17 for men and ≥ 23 for women). The proportion of UPF in the diet was computed for each subject using the NOVA classification applied to dietary intakes collected by repeated 24-h records (mean = 8; SD = 2.3). The association between UPF and depressive symptoms was evaluated using multivariable Cox proportional hazards models.

**Results:**

Over a mean follow-up of 5.4 years, 2221 incident cases of depressive symptoms were identified. After accounting for a wide range of potential confounders, an increased risk of depressive symptoms was observed with an increased %UPF in the diet. In the main model adjusted for sociodemographic characteristics, body mass index, and lifestyle factors, the estimated hazard ratio for a 10% increase in UPF was 1.21 (95% confidence interval = 1.15–1.27). Considering %UPF in food groups, the association was significant only for beverages and sauces or added fats.

**Conclusion:**

Overall, UPF consumption was positively associated with the risk of incident depressive symptoms, suggesting that accounting for this non-nutritional aspect of the diet could be important for mental health promotion.

**Electronic supplementary material:**

The online version of this article (10.1186/s12916-019-1312-y) contains supplementary material, which is available to authorized users.

## Introduction

Depression is a very common disorder, one of the five leading causes of years lived with disability in 2016 [1] and, according to WHO, the 1st leading cause of disease burden globally [2]. Depression etiology implies complex interactions between various factors including social, psychological, and biological factors.

Some treatments are effective but their limitations, as well as the detrimental effect of any depressive episode on the future course of the disease, make prevention crucial [3]. Among large-scale preventive interventions, acting on modifiable factors such as diet is a good candidate for public health action. Large-scale epidemiological studies have consistently documented an association between a healthy diet or dietary indexes reflecting the holistic quality of the diet and a lower risk of depression [4–7]. For instance, in the NutriNet-Santé study, we have observed that several dietary indexes reflecting nutritional recommendations were prospectively and inversely associated with the risk to develop depressive symptoms [8]. On the opposite, a western dietary pattern or pro-inflammatory diet characterized among other things by more processed foods has been associated with poor mental health [5, 6, 9]. Previous studies that reported associations between these diets and depression considered nutritional characteristics of the diet and interaction within the food matrix. However, some of those diets integrate a large part of ultra-processed food (UPF) (i.e., industrial recipes that are practical, ready to eat, and palatable [10]) which consumption has drastically increased over the past decades [11, 12]. For instance, a recent American study reported that, between 2007 and 2012, about 60% of the overall energy intake was provided by UPF [13]. In the French NutriNet-Santé study, UPF contributed to 35.9% of the daily energy intake and the proportion of UPF (%UPF) in the diet has been associated with a poor overall quality of the diet [14].

While processing ensures improvement of food availability, digestibility, short-term safety, transportability, and storage life [15], UPF are often energy-dense; mostly very rich in fat, sugar, and salt; and poor in micronutrients; thus, they may have a potential deleterious role on health. Beyond their unfavorable nutritional composition, they also contain other components generated during transformation such as neo-formed molecules produced during heating, food additives used in manufacturing, and molecules migrated from packaging, some of which might have a detrimental role for gut microbiota [16], involved in the development of several diseases characterized by an inflammatory component (including depression) [17]. The investigation of the association between UPF consumption and health is therefore important.

Recent studies on the link between UPF consumption and health have shown a positive association between UPF consumption and obesity [18], hypertension [19], metabolic disorders [20], and cancer [21]. To date, no study has focused on mental disorders.

The purpose of the present study was thus to investigate for the first time the prospective association between %UPF in the diet and the risk of depressive symptoms using the data of the NutriNet-Santé cohort study.

## Methods

### Study population

The data used in the current study are based on the web-based observational NutriNet-Santé cohort study, launched in France in 2009. The objective of the study is to investigate the link between nutrition and health, as well as determinants of dietary behaviors and nutritional status. Details on the design and method have been previously described [22]. Participants are adult volunteers (aged ≥ 18 years) recruited from the general population (all regions of France) with access to Internet by a vast multimedia campaign. Yearly, participants are asked to complete a set of self-administered web-based questionnaires related to sociodemographic data, economic conditions, physical activity, dietary intake, anthropometric data, and health status. The NutriNet-Santé study is conducted in accordance with the Declaration of Helsinki and was approved by the ethics committee of the French Institute for Health and Medical Research (IRB Inserm no. 0000388FWA00005831) and by the National Commission on Informatics and Liberty (CNIL no. 908450 and no. 909216). Electronic informed consent was obtained from all participants. The NutriNet-Santé study is registered in ClinicalTrials.gov (NCT03335644).

### Depressive symptoms

Two years after inclusion and every 2 years thereafter, depressive symptoms were assessed using the French version of the validated self-administered Center for Epidemiologic Studies Depression (CES-D) scale [23, 24]. The CES-D scale is composed of 20 items evaluating the frequency of depressive symptoms during the previous week. Response modalities are based on a four-point scale (0 = ‘less than 1 day’, 1 = ‘1–2 days’; 2 = ‘3–4 days’; and 3 = ‘5–7 days’). All sub-scores were summed to yield a total score ranging from 0 (no depressive symptoms) to 60 (elevated depressive symptoms). In our study, the internal consistency assessed by Cronbach’s alpha coefficient was high (> 0.80) at each CES-D scale assessment. In the present study, the presence of depressive symptoms was defined using the French validated cut-off values (CES-D ≥ 17 in men and ≥ 23 in women) [23, 24]. We defined ‘incident cases of depressive symptoms’ as participants who were free of depressive symptoms at the 1st CES-D assessment and then presenting depressive symptoms at least once during follow-up (i.e., based on one or multiple of the CES-D questionnaires completed after the initial CES-D assessment).

### Dietary data and ultra-processed food consumption assessment

At inclusion and every 6 months thereafter, participants were invited to provide three non-consecutive 24-h dietary records. These were randomly assigned over a 2-week period (two weekdays and one weekend day) to cover intra-individual variability in intake. Consumption of all types of foods and beverages were reported on the web-based dietary record platform validated for self-administration [25]. The NutriNet-Santé web-based self-administered 24-h dietary records have also been validated against blood and urinary biomarkers [26, 27]. Portion sizes were determined using validated photographs [28] and household measures or directly by providing exact quantity (grams/milliliters). Energy and nutrient intakes were estimated using the published NutriNet-Santé food composition table including more than 3000 food items [29]. Composite home-made dishes were decomposed by using French recipes validated by nutrition professionals. Dietary under-reporters were identified using the method developed by Black [30]. The dietary data used in the present study are those collected during the first 2 years of follow-up (inclusion until the first CES-D assessment). Daily mean food intakes were calculated from all dietary records weighted according to the type of day (weekdays or weekend) with, on average, 7.98 (SD = 2.28) recorded days.

To account for the dietary profiles of participants, as a potentially strong confounder in the context of our study, we used principal component analysis (PCA) to extract ‘dietary pattern scores’ that are independent linear combinations of 22 pre-defined food groups, maximizing the explained variance. The number of dietary patterns retained was determined according to Cattel’s Scree plots and the interpretability of the principal components. Food groups with absolute loading coefficient > 0.3 were considered to be strongly associated with a pattern, and an individual pattern score was calculated by summing the intake of the 22 food groups, weighted by their loading coefficients. The first two dietary patterns accounted for about 18% of the initial variance (Additional file 1: Table S1). The first principal component, corresponding to a “healthy” dietary pattern, was strongly and positively correlated with intake of whole grains, olive oil, vegetables, and fruit. The second principal component, corresponding to a “western” dietary pattern, was strongly correlated with refined grains, potatoes, meat, and alcoholic beverages.

### Classification of the level of processing

All foods and beverages were classified according to the four-group NOVA food classification system (un/minimally processed, culinary ingredient, processed food, and ultra-processed food) [12, 31]. The present study primarily focused on the ‘ultra-processed foods’ (UPF) category. The proportion (in weight, % grams/day) of UPF (%UPF) in the diet was calculated for each participant. UPF are manufactured food products containing numerous ingredients as well as additives such as hydrogenated oils, non-sugar sweeteners, modified starch, flavoring agents, emulsifiers, humectants, colors, and other additives used for cosmetic purpose. This food category includes among others: mass-produced packaged breads and buns; breakfast ‘cereals’, and ‘energy’ bars; sweet or savory packaged snacks; carbonated and ‘energy’ drinks; sweet fruit-based desserts with added sugars, artificial flavours and texturizing agents; flavoured milk drinks and cocoa drinks; industrial cookies, pastries, cakes, and cake mixes; confectionery (ice-cream, chocolate, candies); meat and chicken extracts and ‘instant’ sauces; margarines and spreads; cooked seasoned vegetables with ready-made sauces; ready-to-heat products (powdered and packaged ‘instant’ soups, noodles and desserts, pre-prepared pies, pasta and pizza dishes, poultry and fish ‘nuggets’, burgers, hot dogs, and other reconstituted meat products).

### Covariates

Data on sex, date of birth, marital status (living alone, cohabiting, or separated/divorced/widowed), educational level (less than high school diploma, high school diploma, or university level), occupational categories (never-employed/other activity, self-employed, employee, intermediate profession, and managerial staff), residential area (rural or urban), smoking status (never, former or current smoker), household composition, and monthly household income (< 1200, 1200–1800, 1800–2700, > 2700 euros and a category of participants who refused to disclose their income) were collected at baseline using a self-administered web-based questionnaire [32].

Monthly household income was estimated per consumption unit (CU) using a weighting system: one CU attributed for the first adult in the household, 0.5 CU for other persons aged 14 or older, and 0.3 CU for children under 14 [33]. Weight and height data were collected by a validated self-administered anthropometric questionnaire [34]. Body mass index (BMI) was calculated as the ratio of weight to squared height (kg/m^2^). Physical activity was assessed using a short form of the French version of the International Physical Activity Questionnaire (IPAQ) [35]. Energy expenditure was classified as low physical activity (< 30 min of physical activity; equivalent to brisk walking/day), moderate physical activity (≥ 30 and < 60 min) or high physical activity (≥ 60 min). Prevalent and incident cases of cancer and cardiovascular diseases (strokes, myocardial infarctions, and acute coronary syndromes) were self-reported during follow-up; incident cases were validated by a medical committee based on medical records (diagnosis, hospitalization, radiological reports, electrocardiograms, etc.), and a link was made with medico-administrative databases of the French National Health insurance. Type 2 diabetes and hypertension were self-reported or identified using specific medication. In addition, subjective memory complaints were measured concomitantly with depressive symptoms scale using the French version of the validated self-administered Cognitive Difficulties Scale (CDS) [36, 37].

### Statistical analysis

**Fig. 1:**
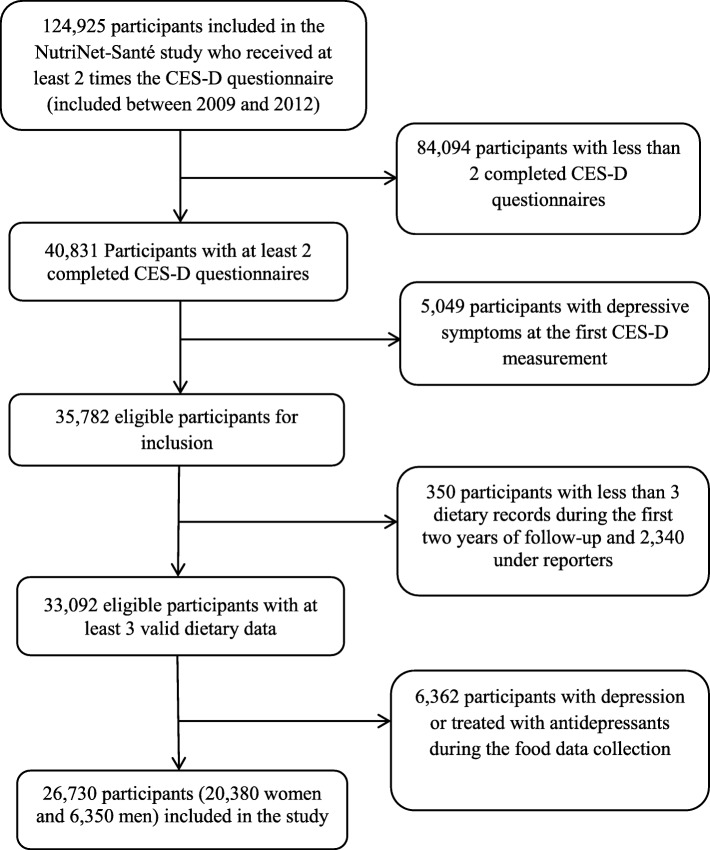
Flow chart of participant selection. *CES-D* Center for Epidemiologic Studies Depression Scale

The inclusion criteria in this study were as follows: (1) have received at least twice the CES-D questionnaire (included between 2009 and 2012), (2) completed at least two of these questionnaires, and (3) not present depressive symptoms at the first CES-D assessment. Among the participants who met these criteria (*n* = 35,782), we excluded participants without valid dietary data (participants with less than three dietary records during the first 2 years of follow-up and under-reporters) and the participants who had reported depression or treatment with antidepressants during the dietary data collection. Thus, a final study sample of 26,730 participants was obtained (Fig. 1).

In the present study, data were missing for some covariates (*n* = 7 for marital status, *n* = 72 for occupational categories, *n* = 317 for residential area, *n* = 195 for educational level, and *n* = 435 for physical activity). As the proportion of missing values was < 1%, they were handled using the Hot Deck method, i.e., by replacing missing values with the value of respondents with similar characteristics [38] .

Participants included in the present study were compared with excluded eligible participants using chi-square tests or *t* tests. Participants’ characteristics and nutritional factors were compared across quartiles of %UPF using linear contrast or Cochran-Mantel-Haenszel tests. For descriptive purposes, nutrient intakes were energy-adjusted using the residual method [39].

The associations between %UPF (modeled as quartiles and as a continuous variable, while estimating coefficients associated with a 10% increase in UPF) and risk of depressive symptoms were assessed using Cox proportional hazards regression models for interval censored data. Hazard ratios (HR) and 95% confidence intervals (CI) were estimated. Linear trend tests across quartiles of %UPF were assessed by modeling these quartiles as ordinal variables. Age was used as the primary time scale variable. Entry time was defined as the age at the first CES-D measurement. Exit time was the age at last completed CES-D questionnaire, or the average of the age between the first occurrence of depressive symptoms and the age at the previous measurement for non-cases and cases respectively.

The first model was adjusted for age, sex, and BMI (continuous variable). The second model (main model) was additionally adjusted for marital status, educational level, occupational categories, monthly household income per consumption unit, residential area, energy intake without alcohol, number of 24 h records and inclusion month, smoking status, alcohol consumption, and physical activity. Five additional models were also performed to account for (a) PCA-extracted dietary patterns and intake of carbohydrates, lipids, and salt; (b) health events occurring during follow-up (cancer, type 2 diabetes, and cardiovascular diseases); (c) baseline CES-D score (continuous variable), and use of antidepressants during the follow-up; (d) CDS score (continuous variable), and (e) baseline CES-D score (continuous variable), use of antidepressants during the follow-up and CDS score (continuous variable).

A potential interaction between %UPF and the Western and the healthy dietary patterns on the risk of depressive symptoms was tested. We also considered a potential interaction between %UPF and the ratio between energy intake and energy needs calculated using the PAL (physical activity level) and basal metabolic rate (which was estimated using the Schofield equations accounting for age, sex, weight, and height [40]).

Another supplementary analysis was performed by considering the % of UPF within each food group. For this analysis, models were further adjusted for the intake of the considered food group. To account for the multiple testing, false discovery rate-corrected *P* values were estimated using the Benjamini-Hochberg procedure [41].

### Sensitivity analyses

A number of sensitivity analyses were performed to test the robustness of our findings. First, for comparison with international data, the proportion of total energy intake from UPF, usually used in other studies, in the diet was also calculated and the main analyses were rerun. Second, we tested the robustness of our findings when other CES-D cut-offs (a) 16 or (b) 19 were considered [23, 24]. We also repeated the analyses by considering as cases, only the participants who had depressive symptoms during follow-up (according to CES-D score) and also reported antidepressant treatment during follow-up. All statistical analyses were conducted using SAS (version 9.4; SAS institute Inc., Cary, NC, USA) with a significance level of 0.05 for two-sided tests.

## Results

In the NutriNet-santé cohort, participants who completed only 1 CES-D questionnaire (*n* = 24,154), compared to those who completed it at least two during follow-up (*n* = 40,831) were younger, less physically active, and more likely to be women, current smoker or living alone. They were also more likely to have a BMI value ≥ 30, a slightly higher baseline CES-D score, a household income per unit consumption < 1800, or not having provided their income and less likely to have a chronic disease and an intermediate profession or to be managerial staff (Additional file 2: Table S2). In addition, among eligible participants (*n* = 35,782), those included were more educated, more often managerial staff, and more often physically active and presented less often an obesity or chronic diseases than those excluded (Additional file 3: Table S3).

The study sample included 6350 men and 20,380 women with a mean age of 47.26, standard deviation (SD = 14.17) years at baseline. During follow-up (mean = 5.4, SD = 1.13 years), a total of 2221 incident cases of depressive symptoms (9.0% in men and 8.1% in women; *p* = 0.02) were identified. Baseline characteristics of the studied sample are presented in Table 1.

**Table 1:** Baseline characteristics according to the quartiles of ultra-processed food (UPF) consumption, NutriNet-Santé study (*n* = 26,730)

Baseline characteristics	Quartile 1	Quartile 2	Quartile 3	Quartile 4	*P* trend^a^
%UPF, range	0%–10%	10%–14%	14%–19%	19%–76%	
%UPF, median (IQR)	7% (3%)	12% (2%)	16% (2%)	23% (8%)	
*n*	6682	6683	6683	6682	
Age, year	51.6 ± 12.2	48.9 ± 13.4	46.6 ± 14.2	42.0 ± 15.0	< 0.0001
Sex, *n* (%)					0.43
Male	1520 (22.7)	1663 (24.9)	1577 (23.6)	1590 (23.8)	
Female	5162 (77.3)	5020 (75.1)	5106 (76.4)	5092 (76.2)	
Marital status, *n* (%)					< 0.0001
Living alone	619 (9.2)	777 (11.6)	920 (13.8)	1332 (19.9)	
Cohabiting	5244 (78.5)	5202 (77.9)	5093 (76.2)	4785 (71.6)	
Separated/divorced/widowed	819 (12.3)	704 (10.5)	670 (10.0)	565 (8.5)	
Educational level, *n* (%)					0.29
< High school diploma	1311 (19.6)	1283 (19.2)	1369 (20.5)	1226 (18.3)	
High school diploma	986 (14.8)	964 (14.4)	983 (14.7)	1196 (17.9)	
University level	4385 (65.6)	4436 (66.4)	4331 (64.8)	4260 (63.8)	
Occupational categories, n (%)					< 0.0001
Never-employed/other activity	103 (1.5)	150 (2.2)	227 (3.4)	367 (5.5)	
Self employed	338 (5.1)	324 (4.9)	316 (4.7)	373 (5.6)	
Employee	1369 (20.5)	1479 (22.1)	1680 (25.2)	1975 (29.6)	
Intermediate profession	1984 (29.7)	2022 (30.3)	1973 (29.5)	1834 (27.4)	
Managerial staff	2888 (43.2)	2708 (40.5)	2487 (37.2)	2133 (31.9)	
Household income, *n* (%)					< 0.0001
Not answered	664 (9.9)	587 (8.8)	654 (9.8)	744 (11.1)	
< 1200 euros	624 (9.3)	722 (10.8)	825 (12.3)	1071 (16.0)	
1200–1800 euros	1349 (20.2)	1516 (22.7)	1623 (24.3)	1698 (25.4)	
1800–2700 euros	1668 (25.0)	1717 (25.7)	1727 (25.8)	1712 (25.6)	
≥ 2700 euros	2377 (35.6)	2141 (32.0)	1854 (27.7)	1457 (21.8)	
Residential area, *n* (%)					0.07
Rural	1431 (21.4)	1444 (21.6)	1519 (22.7)	1499 (22.4)	
Urban	5251 (78.6)	5239 (78.4)	5164 (77.3)	5183 (77.6)	
Smoking status, *n* (%)					< 0.0001
Former smoker	2799 (41.9)	2547 (38.1)	2313 (34.6)	2071 (31.0)	
Current smoker	721 (10.8)	809 (12.1)	771 (11.5)	905 (13.5)	
Never-smoker	3162 (47.3)	3327 (49.8)	3599 (53.9)	3706 (55.5)	
Physical activity, *n* (%)^b^					< 0.0001
Low	1212 (18.1)	1473 (22.1)	1682 (25.2)	2014 (30.1)	
Moderate	1478 (22.1)	1612 (24.1)	1660 (24.8)	1614 (24.2)	
High	3992 (59.8)	3598 (53.8)	3341 (50.0)	3054 (45.7)	
Body mass index^c^, *n* (%)					0.001
Underweight	294 (4.4)	276 (4.1)	272 (4.1)	350 (5.2)	
Normal weight	4517 (67.6)	4459 (66.7)	4417 (66.1)	4323 (64.7)	
Overweight	1448 (21.7)	1477 (22.1)	1522 (22.8)	1414 (21.2)	
Obesity	423 (6.3)	471 (7.1)	472 (7.0)	595 (8.9)	
Chronic diseases^d^, *n* (%)	741 (11.1)	724 (10.9)	683 (10.2)	559 (8.4)	< 0.0001
Baseline CES-D, mean score	7.74 ± 5.38	7.94 ± 5.35	8.26 ± 5.46	8.90 ± 5.56	< 0.0001

*CES-D* Center for Epidemiologic Studies Depression Scale, *UPF* Proportion of ultra-processed food intake

Values are means ± standard deviation or numbers (percentages) as appropriate

^a^*P* trend values are based on linear contrast or Cochran-Mantel-Haenszel tests

^b^Physical activity was classified as low (< 30 min of physical activity; equivalent to brisk walking/day) or moderate/high physical activity (≥ 30 min of physical activity; equivalent to brisk walking/day)

^c^Body mass index was classified as underweight (BMI < 18.5), normal weight (18.5 ≥ BMI < 25), overweight (25 ≥ BMI < 30) or obese (BMI ≥ 30)

^d^Incident cases of cancer, type 2 diabetes, hypertension and cardiovascular diseases

The average %UPF was 15% (SD = 8%) in gram and 32% (SD = 11%) in energy. Participants with an elevated %UPF in the diet were younger, more often employees, never or current smokers, and had a lower income. They also more often presented obesity. The %UPF in the diet was also associated with a less nutritionally healthy diet (Table 2), since higher %UPF values were associated with higher energy intakes and saturated fatty acids intakes. On the other hand, a negative correlation with micronutrients (beta-carotene, vitamin C, folic acid, Vitamin B12, magnesium, and fibers) and omega 3 fatty acids was observed.

**Table 2:** Baseline nutritional and dietary intakes according to the quartiles of ultra-processed food consumption, NutriNet-Santé study

Nutritional factors	Quartile 1	Quartile 2	Quartile 3	Quartile 4	*P* trend^a^
UPF, range	0%–10%	10%–14%	14%–19%	19%–76%	
UPF, median (IQR)	7% (3%)	12% (2%)	16% (2%)	23% (8%)	
n	6682	6683	6683	6682	
Total energy intake, Kcal/d	1830 ± 434	1913 ± 446	1921 ± 448	1934 ± 459	< 0.0001
Alcohol intake, g/d	9.5 ± 12.2	9.8 ± 12.2	8.3 ± 10.7	6.9 ± 9.9	< 0.0001
Energy intake without alcohol, Kcal/d	1764 ± 413	1845 ± 421	1863 ± 427.4	1886 ± 442	< 0.0001
Carbohydrates,% energy^b^	42.8 ± 6.5	43.1 ± 5.7	43.2 ± 5.7	43.6 ± 5.7	< 0.0001
Lipids, % energy^b^	38.2 ± 6.1	38.7 ± 5.4	38.9 ± 5.1	39.0 ± 5.3	< 0.0001
Saturated fatty acids, g/d^c^	31.6 ± 6.7	32.8 ± 6.5	33.4 ± 6.3	33.7 ± 6.4	< 0.0001
Monounsaturated fatty acids, g/d^c^	30.8 ± 6.7	30.4 ± 5.8	30.3 ± 5.6	30.2 ± 5.5	< 0.0001
Polyunsaturated fatty acids, g/d^c^	11.6 ± 3.9	11.4 ± 3.4	11.3 ± 3.3	11.4 ± 3.4	0.003
Omega-3 fatty acids, g/d^c^	1.6 ± 0.7	1.4 ± 0.6	1.4 ± 0.6	1.3 ± 0.6	< 0.0001
Protein, % energy^b^	18.6 ± 3.8	17.8 ± 3.2	17.6 ± 3.3	17.1 ± 3.4	< 0.0001
Beta-carotene, μg/d^c^	4031 ± 2233	3668 ± 1845	3502 ± 1927	3121 ± 1893	< 0.0001
Vitamin C, mg/d^c^	132 ± 58.9	122 ± 63.0	116 ± 63.0	107 ± 64.1	< 0.0001
Vitamin D, μg/d^c^	2.9 ± 1.8	2.8 ± 1.6	2.7 ± 1.5	2.5 ± 1.5	< 0.0001
Vitamin E, mg/d^c^	12.1 ± 3.6	11.7 ± 3.2	11.6 ± 3.2	11.5 ± 3.3	< 0.0001
Folic acid, μg/d^c^	356 ± 91.5	337.9 ± 79.7	330 ± 82.5	311 ± 86.3	< 0.0001
Vitamin B12, μg/d^c^	5.8 ± 4.4	5.5 ± 3.7	5.3 ± 3.7	5.0 ± 3.5	< 0.0001
Magnesium, mg/d^c^	367 ± 88.0	348 ± 80.3	334 ± 81.7	318 ± 86.8	< 0.0001
Fiber (g/d)^c^	22.0 ± 5.7	20.6 ± 5.1	19.7 ± 5.1	18.2 ± 5.6	< 0.0001
Starchy foods	213 ± 95.8	209 ± 92.2	199 ± 86.6	182 ± 83.8	< 0.0001
Fruit and vegetables	579 ± 241	530 ± 215	503 ± 215	450 ± 225	< 0.0001
Meat, fish, eggs	140 ± 64.1	134 ± 59.8	130 ± 59.8	120 ± 61.7	< 0.0001
Alcoholic drinks	113 ± 151	118 ± 150	99.1 ± 129	82.9 ± 118	< 0.0001
Beverages	1385 ± 580	1232 ± 481	1092 ± 438	945 ± 405	< 0.0001
Dairy products	231 ± 152	233 ± 137	245 ± 139	256 ± 146	< 0.0001
Fatty / sweet products	80.2 ± 51.1	99.0 ± 55.1	106 ± 58.7	116 ± 63.1	< 0.0001
snacks	104 ± 66.4	125 ± 69.5	138 ± 75.4	156 ± 86.8	< 0.0001
Sauces/added fats	28.7 ± 16.5	28.1 ± 16.1	27.4 ± 16.5	25.5 ± 16.7	< 0.0001

*UPF* Proportion of ultra-processed food intake

Values are means ± standard deviation

^a^*P* trend values are based on linear contrast

^b^Values are percentages of total daily energy intake (without alcohol)

^c^Values were adjusted for energy intake without alcohol using the residual method

The associations between ultra-processed food intake and incident depressive symptoms are presented in Table 3. In the main model adjusted for sociodemographic and lifestyle data, a strong and linear relationship was observed between %UPF in the diet and the risk of incident depressive symptoms. A 10% increase in %UPF in the diet was associated with a 21% (95%CI = 15%–27%) higher risk of depressive symptoms. Further adjustment for dietary patterns and dietary intakes (carbohydrates, lipids, and sodium), health events, or the CDS score did not substantially modify the association. However, after accounting for the use of antidepressants during follow-up and the baseline value for the CES-D score, the association was attenuated but remained significant (HR_for a 10% increase in UPF in the diet_ = 1.14, 95%CI = 1.09–1.20). The proportional hazards assumption was evaluated using martingale residues, and the assumption was acceptable (*P* = 0.12 for the main model). The dose-response association between %UPF and incident depressive symptoms using Restricted Cubic Spline was also presented in Additional file 4: Figure S1 (*p* < 0.0001 for the overall association).

**Table 3:** Association between ultra-processed food intake and incident depressive symptoms, NutriNet-Santé study

	Quartile 1	Quartile 2	Quartile 3	Quartile 4	*P* trend	Continuous^a^	*P* ^b^
UPF, range	0%–10%	10%–14%	14%–19%	19%–76%			
UPF, median (IQR)	7% (3%)	12% (2%)	16% (2%)	23% (8%)			
*n*	6682	6683	6683	6682		26,730	
Number of cases	491	459	557	714		2221	
Person years	21,597	21,097	20,468	19,918		83,080	
Model 1^c^	1 (ref)	0.90 (0.79; 1.02)	1.07 (0.94; 1.21)	1.31 (1.16; 1.47)	< 0.0001	1.23 (1.17; 1.29)	< 0.0001
Model 2^d^	1 (ref)	0.91 (0.80; 1.04)	1.09 (0.96; 1.23)	1.30 (1.15; 1.47)	< 0.0001	1.21 (1.15; 1.27)	< 0.0001
Model 3^e^	1 (ref)	0.91 (0.80; 1.04)	1.08 (0.95; 1.23)	1.29 (1.13; 1.47)	< 0.0001	1.22 (1.16; 1.29)	< 0.0001
Model 4^f^	1 (ref)	0.92 (0.81; 1.04)	1.09 (0.97; 1.24)	1.31 (1.16; 1.48)	< 0.0001	1.21 (1.15; 1.27)	< 0.0001
Model 5^g^	1 (ref)	0.88 (0.77; 1.00)	1.00 (0.88; 1.13)	1.13 (1.00; 1.28)	0.01	1.14 (1.09; 1.20)	< 0.0001
Model 6^h^	1 (ref)	0.88 (0.78; 1.00)	1.06 (0.94; 1.20)	1.27 (1.13; 1.44)	< 0.0001	1.21 (1.15; 1.27)	< 0.0001
Model 7^i^	1 (ref)	0.86 (0.76; 0.98)	1.00 (0.88; 1.13)	1.13 (1.00; 1.28)	0.01	1.15 (1.09; 1.21)	< 0.0001

Values are hazard ratios (95% confidence intervals).*CDS* Cognitive Difficulties Scale; *CES-D* Center for Epidemiologic Studies Depression Scale; *IQR* interquartile range; *UPF* proportion of ultra-processed food intake

^a^Hazard ratios for 10% increase in the proportion of ultra-processed food intake

^b^*P* for continuous variable

^c^Adjusted for age, sex, and body mass index

^d^Adjusted for all variables in model 1 + marital status, educational level, occupational categories, household income per consumption unit, residential area, number of 24-h dietary records, inclusion month, energy intake without alcohol, alcohol intake, smoking status, and physical activity (main model)

^e^Adjusted for all variables in model 2 + dietary patterns derived from the factor analysis (‘Healthy’ and ‘Western’ dietary pattern) and intakes of lipids, sodium, and carbohydrates

^f^Adjusted for all variables in model 2 + health events during follow-up (cancer, type 2 diabetes, hypertension and cardiovascular events)

^g^Adjusted for all variables in model 2 + use of antidepressants during follow-up and baseline CES-D score

^h^Adjusted for all variables in model 2 + CDS score

^i^Adjusted for all variables in model 2 + use of antidepressants during follow-up, baseline CES-D score, and CDS score

**Fig. 2:**
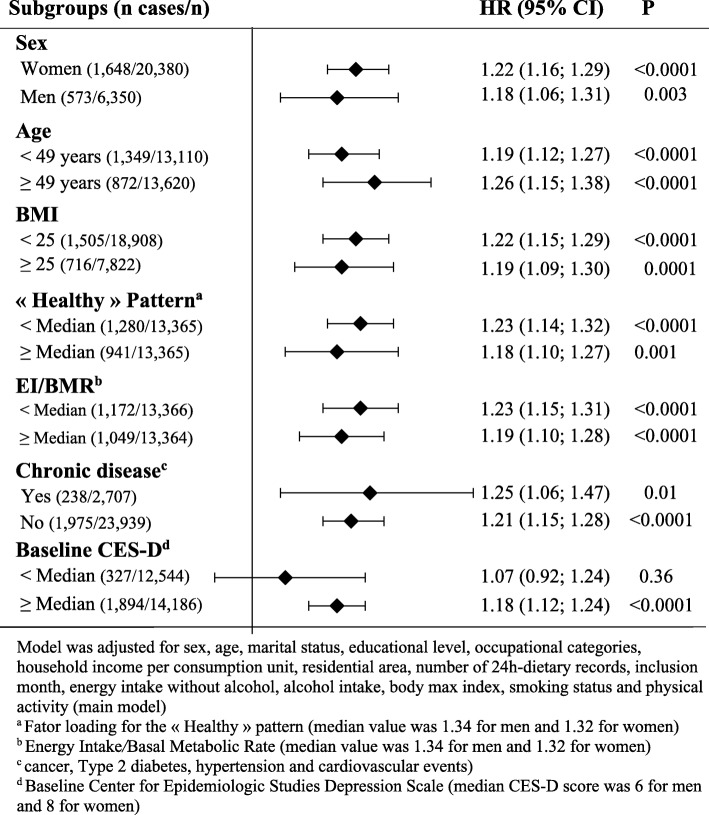
Association between ultra-processed food intake and incident depressive symptoms in population subgroups. Values are hazard ratios (HR) and 95% confidence intervals (95% CI). *BMI* body max index; *BMR* basal metabolic rate; *CES-D* Center for Epidemiologic Studies Depression Scale; *EI* energy intake. Model was adjusted for sex, age, marital status, educational level, occupational categories, household income per consumption unit, residential area, number of 24-h dietary records, inclusion month, energy intake without alcohol, alcohol intake, body max index, smoking status, and physical activity (main model)

On the other hand, when considering the ratio of energy intake to energy needs, a significant interaction with %UPF was detected (*P* = 0.04). Hence, analysis stratified according to this ratio (while using the sex-specific EI/BMR median value as cut-off: 1.34 for men and 1.32 for women) was performed (Fig. 2). The association between %UPF and the risk of depressive symptoms was stronger in participants with low energy intakes compared to their needs than their counterparts (HR_for a 10% increase in UPF in the diet_ = 1.23, 95%CI = 1.15–1.31 versus 1.19, 95%CI = 1.10–1.28; main model). Similarly, in the stratified analyses according to sex, age (using the median value), BMI (< 25 vs. ≥ 25), comorbid conditions and individual pattern score for the ‘Healthy’ pattern (using the sex-specific median value as cut-off: 0.04 for men and − 0.11 for women), stronger associations were observed in the subgroups of women, participants aged ≥ 49 years, participants with a chronic disease, participants with a BMI value ≥ 25, and participants with a lower value for the individual score of the ‘Healthy’ pattern, compared to their respective counterparts (Fig. 2). However, the estimated HR were similar in the subgroups and no significant interaction was observed (*P* value for the interaction term was 0.49 for sex, 0.46 for age, 0.59 for BMI, 0.88 for comorbidities, and 0.18 for the individual score of the ‘healthy’ dietary pattern). In addition, in the stratified analyses according to the baseline CES-D score (using the sex-specific median value as cut-off: 6 for men and 8 for women), the association between %UPF and the risk of depressive symptoms was significant only among participants with a higher baseline CES-D score (*P* value for the interaction term was 0.13).

The associations between %UPF in each food group and the risk of depressive symptoms are presented in Table 4. A significantly increased risk was observed for high %UPF in beverages and sauces or added fats. The strongest association was detected for %UPF in beverages (HR_Q4 vs.Q1_ = 1.25, 95%CI = 1.13–1.38).

**Table 4:** Association between the % of ultra-processed in each food group and incident depressive symptoms, NutriNet-Santé study, *N* = 26,730

	Quartile 1	Quartile 2	Quartile 3	Quartile 4	*P* trend^a^
Starchy foods	1 (ref)	0.97 (0.86; 1.10)	0.97 (0.86; 1.10)	1.01 (0.89; 1.14)	0.98
Fruit and vegetables	1 (ref)	0.92 (0.81; 1.03)	0.97 (0.86; 1.10)	1.08 (0.95; 1.22)	0.57
Meat, fish, eggs	1 (ref)	1.08 (0.96; 1.22)	0.97 (0.86; 1.10)	1.04 (0.92; 1.17)	0.98
Beverages	1 (ref)	1.19 (0.91; 1.54)	1.00 (0.89; 1.12)	1.25 (1.13; 1.38)	0.002
Dairy products	1 (ref)	1.03 (0.91; 1.16)	1.06 (0.94; 1.20)	1.13 (1.00; 1.27)	0.20
Fatty/sweet products	1 (ref)	1.02 (0.90; 1.16)	1.05 (0.93; 1.18)	1.08 (0.96; 1.22)	0.57
Snacks	1 (ref)	0.97 (0.85; 1.10)	1.10 (0.98; 1.25)	1.10 (0.98; 1.24)	0.18
Sauces/added fats	1 (ref)	1.05 (0.93; 1.19)	0.96 (0.85; 1.09)	1.23 (1.10; 1.39)	0.02

Values are hazard ratios (95% confidence intervals) and linear trend tests across the quartiles were assessed by modeling the quartiles of %UPF as ordinal variables. *UPF* Proportion of ultra-processed food intake

Models are adjusted for age, sex, marital status, educational level, occupational categories, household income per consumption unit, residential area, energy intake without alcohol, number of 24-h dietary records, inclusion month, smoking status, physical activity, body mass index, health events during follow-up (cancer, type 2 diabetes, hypertension and cardiovascular events) and quantity of the equivalent food group

^a^Corrected using the Benjamini-Hochberg procedure

### Sensitivity analyses

Sensitivity analyses using other CES-D cut-offs (16 or 19) to identify cases of depressive symptoms or %UPF weighted on energy intake rather than on quantity consumed in gram yielded similar associations (Additional file 5: Table S4 and Additional file 6: Table S5). In addition, the analyses considering as cases only participants who had depressive symptoms and also used antidepressant treatment during follow-up showed stronger associations (Additional file 7: Table S6). However, the associations were not significant when the %UPF was modeled as quartiles, mainly due to low statistical power because of the small number of cases (*n* = 113 cases). In the main model, the estimated HR for the analysis with a 10% increase in UPF consumption was 1.43 (95%CI = 1.18–1.73).

## Discussion

In this large cohort study of adults, the %UPF in the diet was positively associated with the risk of incident depressive symptoms even after extensive adjustment in particular for dietary patterns correlated to %UPF. Indeed, in coherence with previous studies, we found that the %UPF in the diet varied according to the socioeconomic profile and lifestyle of individuals [42, 43].

The first hypothesis which may explain our findings relies on the fact that ultra-processed foods are often part of generally “unhealthy”/western dietary patterns. Although not entirely composed of UPF, western diet is marked by elevated consumption of UPF and has been associated with depressive outcomes in epidemiologic study. Indeed, in a previous investigation based on data from the NutriNet-Santé study, the diet of high consumers of UPF was relatively ‘unhealthy’ [14], i.e., characterized by a low consumption of fruit and vegetables and a high intake of sweet products or soft drinks. Similar findings were observed in a study conducted within the NHANES, a representative survey conducted in the American population [44]. This is of high importance since ‘western’-style dietary patterns have been previously related to depression [5, 6]. In particular, a recent meta-analysis including 21 studies conducted in 10 countries reported that a diet rich in red meat, processed meat, refined grains, sweets, high-fat dairy products, butter, potatoes, and high-fat gravy was associated with an elevated risk of depression: presenting a high versus a low ‘Western-type diet’ score was associated with an 18% (95%CI = 5%–34%) increased risk [6].

In the Whitehall study, which included middle-aged UK adults, a diet rich in some types of UPF foods such as sweetened desserts, fried food, and processed meat but also refined grains and high-fat dairy products was also associated with higher odds of depressive symptoms (OR_tertile3 vs. tertile1_ = 1.58, 95%CI = 1.11–2.23) [45]. An increased risk of depression was also observed among the participants included in the Seguimiento Universidad de Navarra—University of Navarra Follow-up (SUN) Project, who were in the highest quintile of fast food (hamburgers, sausages, pizza) and processed pastries (muffins, doughnuts, croissants) compared with those in the lowest quintile (HR_quintiles 5 vs. quintiles 1_ = 1.37, 95%CI = 1.02–1.83) [46]. In addition, in the Personality and Total Health (PATH) Through Life Study—a longitudinal community study including 3663 Australian participants from 3 age cohorts (20+; 40+; 60+ years), a higher score concerning an unhealthy dietary pattern characterized by a high consumption of roast meat, sausages, hamburgers, steak, chips, crisps, and soft drinks was an independent predictor of the risk depressive symptoms over time [47]. Anyway these studies do not allow to distinguish the specific role of nutritional profile versus non-nutritional components, part of the western diet, implied in the association with depression.

Then, when stratifying analysis on ‘adequate energy intake’ reflected by the ratio between energy intake and energy needs, a stronger association was observed among participants with lower energy intakes. This may suggest that a limited energy intake associated with a large part of UPF in the diet could limit the intakes of bioactive micronutrients that are beneficial for depression prevention.

Importantly, the link between UPF consumption and depression could be at least partly explained by effect of some non-nutrient components used for or produced during processing. Indeed, UPF often contain products additives (in particular emulsifiers) or molecules resulting from high-temperature heating which may among others cause alterations to the gut microbiota [16], which has been suggested to show important interrelations with mental health [48]. To the best of our knowledge, no investigation in humans has been conducted to explore the specific role of food additives for the risk of depression except concerning artificial sweeteners. Some experimental studies argue for a modulating role of artificial sweeteners, such as aspartame, on neurotransmitters regulation which may lead to symptoms such as mood or depression [49]. However, a recent review based on more than 370 scientific papers reported that data are currently insufficient to conclude [50].

A specific role of UPF on depression, beyond nutritional aspects, may, among others, also rely on changes in microbiota induced by non-nutritive components, in particular by emulsifiers which may provoke gut dysbiosis and mediate inflammatory processes in the gut [51]. In addition, a specific nanoparticle used as, TiO_2_whitening agent, has been related to neuroinflammation in an animal model [52]. Findings from animal studies have suggested that some food additives (e.g., monosodium glutamate) may induce anxiety and depression symptoms [53] or increase susceptibility to the depressor stimuli [54].

The association reported in this study is of interest in terms of public health namely for prevention of depression. In this context, it should be noted that the benefit of decreasing %UPF in diet may be even stronger in other populations than in our sample of French volunteers included in a diet-related study. Indeed, while UPF (as % of energy) accounted for 32% in our population, a higher proportion has been documented in other studies. For instance, in the UK national diet and Nutrition Survey, 53% of the energy intake [55] was provided through UPF. In North America, %UPF was even higher as evaluated by the representative survey (NHANES), with an average of 57.5% of calories coming from ultra-processed foods [44]. Such elevated consumption of UPF may be an important lever in terms of public health strategy for the prevention of depression. Our results showing that the association between %UPF and the risk of depressive symptoms vary across food groups may help guiding future research toward the non-nutrient components that are most likely to convey an increased risk of depression. Should ultra-processed beverages, dairy products, snacks, and fats share common food additives that are less present in other food groups, these food additives might warrant further scrutiny.

Some limitations of our study should be noted. First, the allocation of foods to the categories defined by the NOVA may have led to misclassification bias—particularly since the food composition table used so far in our study is based on generic foods, and not foods as sold. Thus, for food which can be more or less processed, the most frequent level of processing for a food item was applied. Second, given the observational design of our study, we cannot entirely exclude reverse causality, although our study is of prospective nature. Moreover, despite the fact that we accounted for a wide range of confounders in our statistical models, unmeasured factors related to depression such as life events might have led to potential residual confounding; thus, causality of the observed associations is not established. Third, participants of the NutriNet-Santé study were volunteers in a nutrition-related cohort and thus more interested in nutritional issues and healthy lifestyles than the general population. In particular, their consumption of UPF may be lower than in the general population which may have led to an underestimation of the associations investigated in our study. In addition, excluding participants who completed only one CES-D questionnaire and participants with depressive symptoms at baseline might have resulted in excluding those most likely to have depressive symptoms. Similar analysis in this specific population should deserve further investigations. All this might have led to a selection bias and thus a potential bias in the risk estimates. As a result, any generalization of our findings should be done with caution. Important strengths of this study include its prospective design, the large sample, and the repeated assessment of depressive symptoms using a validated tool, as well as the quality of the dietary data based on repeated dietary records allowing to assess usual dietary intakes. Finally, the wide range of confounding factors contributed to improve the validity of our findings.

## Conclusions

In this prospective study, we found a positive association between the %UPF in the overall diet and the risk of incident depressive symptoms. Positive associations were also found for beverages and sauces or added fats, when %UPF in the food groups was investigated.

This study highlights a potential role of non-nutritional aspects of the diet in the depression development. Overall, there is a need to collect more detailed data on the degree of food processing and additive or contaminant contents in food surveys to better explore UPF consumption and its potential impact on health.

## Additional files

Additional file 1: **Table S1.** Loading coefficients of the PCA-extracted dietary patterns. (PDF 13 kb)

Additional file 2: **Table S2.** Comparison of participants who completed one CES-D questionnaire to those who completed it at least two during follow-up, NutriNet-Santé study. (PDF 301 kb)

Additional file 3: **Table S3.** Comparison of included and excluded participants, NutriNet-Santé study. (PDF 132 kb)

Additional file 4: **Figure S1.** Dose-response association between ultra-processed food intake and incident depressive symptoms using Restricted Cubic Spline. (PDF 190 kb)

Additional file 5: **Table S4.** Association between ultra-processed food intake and incident depressive symptoms using other cut-off values to define depressive symptoms, NutriNet-Santé study. (PDF 134 kb)

Additional file 6: **Table S5.** Association between ultra-processed food intake (% of energy) and incident depressive symptoms, NutriNet-Santé study. (PDF 125 kb)

Additional file 7: **Table S6.** Association between ultra-processed food intake and incident depressive symptoms (considering as cases, only the participants who had depressive symptoms and also reported antidepressant treatment during follow-up), NutriNet-Santé study. (PDF 125 kb)

## Abbreviations

BMI: Body mass index (kg/m^2^)
CDS: Cognitive Difficulties Scale
CES-D: Center for Epidemiologic Studies Depression Scale
CI: Confidence interval
CU: Consumption unit
HR: Hazard ratios
IPAQ: International Physical Activity Questionnaire
SD: Standard deviation
UPF: Ultra-processed food
