# Supplementary material for: Prospective association between ultra-processed food consumption and incident depressive symptoms in the French NutriNet-Santé cohort
Source: BMC Med. 2019 Apr 15;17:78. doi: 10.1186/s12916-019-1312-y (PMC6463641; doi:10.1186/s12916-019-1312-y)
Supplement: Supplementary file 1 — Table S1. Loading coefficients of the PCA-extracted dietary patterns. (PDF 13 kb) [file 12916_2019_1312_MOESM1_ESM.pdf]

Supplemental Table 1 Loading coefficients of the PCA-extracted dietary patterns

**Food groups**	**Loading coefficients**	
	**Healthy Pattern**	**Western Pattern**
**% the initial variance**	10.5	7.71
Whole grain products	0.49	
Breakfast cereals		
Non-whole grain products		0.56
Legumes	0.31	
Potatoes		0.57
Other starchy foods/Tubers		
Dairy products		
Olive oil	0.46	
Other vegetable oils		0.41
Broths/Added fat	0.37	0.31
Fatty/salty/sweet products		
Vegetables	0.75	
Vegetable juice		
Fruit	0.58	
Fruit juice		
Meat		0.54
Processed meat		0.41
Fish and seafood	0.36	
Eggs		
Alcoholic drinks		0.42
Sweetened drinks	-0.36	
Unsweetened drinks	0.36	

Only food groups with |loading coefficients| > 0.3 are presented
