# Supplementary material for: Prospective association between ultra-processed food consumption and incident depressive symptoms in the French NutriNet-Santé cohort
Source: BMC Med. 2019 Apr 15;17:78. doi: 10.1186/s12916-019-1312-y (PMC6463641; doi:10.1186/s12916-019-1312-y)
Supplement: Supplementary file 2 — Table S2. Comparison of participants who completed one CES-D questionnaire to those who completed it at least two during follow-up, NutriNet-Santé study. (PDF 301 kb) [file 12916_2019_1312_MOESM2_ESM.pdf]

Supplementary data

Supplemental Table 2 Comparison of participants who completed one CES-D questionnaire to those who completed it at least two during follow-up, NutriNet-Santé study [a]

**Baseline characteristics**	**1 completed CES-D questionnaire**	**At least 2 completed CES-D questionnaires**	**P [b]**
n	24,154	40,831	
**Age, y**	42.2 ± 14.5	47.3 ± 14.1	<0.0001
**Sex, *n* (%)**			<0.0001
Male	4,803 (19.9)	9,820 (24.0)	
Female	19,351 (80.1)	31,011 (76.0)	
**Marital status, *n* (%)**			<0.0001
Living alone	4,516 (18.7)	6,122 (15.0)	
Cohabiting	17,315 (71.7)	30,194 (73.9)	
Separated/Divorced/Widowed	2,323 (9.6)	4,515 (11.1)	
**Educational level, *n* (%)**			<0.0001
< High school diploma	4,610 (19.1)	8,440 (20.7)	
High school diploma	4,084 (16.9)	6,469 (15.8)	
University level	15,460 (64.0)	25,922 (63.5)	
**Occupational categories, *n* (%)**			<0.0001
Never-employed/other activity	1,327 (5.5)	1,351 (3.3)	
Self-employed	1,493 (6.2)	2,192 (5.4)	
Employee	7,473 (30.9)	10,657 (26.1)	
Intermediate profession	6,231 (25.8)	11,676 (28.6)	
Managerial staff	7,630 (31.6)	14,955 (36.6)	
**Household income per consumption unit, *n* (%)**			<0.0001
Not answered	2,943 (12.2)	4,120 (10.1)	
< 1200 euros	4,333 (17.9)	5,476 (13.4)	
1200-1800 euros	6,163 (25.5)	9,674 (23.7)	
1800-2700 euros	5,558 (23.0)	10,266 (25.1)	
≥ 2700 euros	5,157 (21.4	11,295 (27.7)	
**Residential area, *n* (%)**			<0.0001
Rural	5,182 (21.4)	8,917 (21.8)	
Urban	18,972 (78.6)	31,914 (78.2)	
**Smoking status, *n* (%)**			<0.0001
Former smoker	7,942 (32.9)	14,897 (36.5)	
Current smoker	4,189 (17.3)	5,283 (12.9)	
Never-smoker	12,023 (49.8)	20,651 (50.6)	
**Physical activity [c], *n* (%)**			<0.0001
Low	6,750 (27.9)	10,118 (24.8)	
Moderate	5,907 (24.5)	9,691 (23.7)	
High	11,497 (47.6)	21,022 (51.5)	
**Body mass index [d], *n* (%)**			<0.0001
Underweight	1,260 (5.2)	1,940 (4.8)	
Normal weight	15,335 (63.5)	26,225 (64.2)	
Overweight	5,095 (21.1)	9,178 (22.5)	
Obesity	2,464 (10.2)	3,488 (8.5)	
**Prevalence of chronic diseases [e], *n* (%)**	1,068 (4.4)	4,420 (10.9)	<0.0001
**Baseline CES-D score**	12.4 ± 9.4	10.9 ± 8.7	<0.0001

CES-D Center for Epidemiologic Studies Depression Scale
[a] Values are means ± standard deviation or numbers (percentages) as appropriate
[b] P-values are based on T-test or chi-square test
[c] Physical activity was classified as low (< 30 minutes of physical activity; equivalent to brisk walking/day), moderate (30-60 min) and high (≥ 60 min)
[d] Body mass index (BMI) was classified as underweight (BMI < 18.5), normal weight (18.5 < BMI < 25), overweight (25 ≤ BMI < 30) and obesity (BM I ≥ 30)
[e] Incident cases of cancer, Type 2 diabetes, hypertension and cardiovascular diseases
