# Supplementary material for: Prospective association between ultra-processed food consumption and incident depressive symptoms in the French NutriNet-Santé cohort
Source: BMC Med. 2019 Apr 15;17:78. doi: 10.1186/s12916-019-1312-y (PMC6463641; doi:10.1186/s12916-019-1312-y)
Supplement: Supplementary file 3 — Table S3. Comparison of included and excluded participants, NutriNet-Santé study. (PDF 132 kb) [file 12916_2019_1312_MOESM3_ESM.pdf]

Supplemental Table 3 Comparison of included and excluded participants, NutriNet-Santé study [a]

Baseline characteristics	Excluded participants	Included participants	P [b]
**n**	9,052	26,730	
**Age, y**	48.1 ± 13.8	47.3 ± 14.2	<0.0001
**Sex, n (%)**			0.001
Male	2,002 (22.1)	6,350 (23.8)	
Female	7,050 (77.9)	20,380 (76.2)	
**% UPF in the diet**	15% ±8%	15% ±8%	0.47
**Marital status, n (%)**			<0.0001
Living alone	1,388 (15.3)	3,648 (13.7)	
Cohabiting	6,571 (72.6)	20,324 (76.0)	
Separated/Divorced/Widowed	1,093 (12.1)	2,758 (10.3)	
**Educational level, n (%)**			<0.0001
< High school diploma	1,998 (22.1)	5189 (19.4)	
High school diploma	1,431 (15.8)	4129 (15.5)	
University level	5,623 (62.1)	17412 (65.1)	
**Occupational categories, n (%)**			<0.0001
Never-employed/other activity	283 (3.1)	847 (3.2)	
Self-employed	474 (5.2)	1,351 (5.1)	
Employee	2,404 (26.6)	6,503 (24.3)	
Intermediate profession	2,663 (29.4)	7,813 (29.2)	
Managerial staff	3,228 (35.7)	10,216 (38.2)	
**Household income per consumption unit, n (%)**			0.001
Not answered	920 (10.2)	2,649 (9.9)	
< 1200 euros	1,198 (13.2)	3,242 (12.1)	
1200-1800 euros	2,186 (24.1)	6,186 (23.1)	
1800-2700 euros	2,271 (25.1)	6,824 (25.5)	
≥ 2700 euros	2,477 (27.4)	7,829 (29.3)	
**Residential area, n (%)**			0.04
Rural	1,900 (21.0)	5,893 (22.0)	
Urban	7,152 (79.0)	20,837 (78.0)	
**Smoking status, n (%)**			<0.0001
Former smoker	3,341 (36.9)	9,730 (36.4)	
Current smoker	1,239 (13.7)	3,206 (12.0)	
Never-smoker	4,472 (49.4)	13,794 (51.6)	
**Physical activity [c], n (%)**			0.07
Low	2,260 (25.0)	6,368 (23.8)	
Moderate	2,155 (23.8)	6,371 (23.8)	
High	4,637 (51.2)	13,991 (52.4)	
**Body mass index [d], n (%)**			<0.0001
Underweight	431 (4.8)	1,192 (4.5)	
Normal weight	5,638 (62.3)	17,716 (66.3)	
Overweight	2,112 (23.3)	5,861 (21.9)	
Obesity	871 (9.6)	1,961 (7.3)	
**Prevalence of chronic diseases [e], n (%)**	1,059 (11.8)	2,707 (10.2)	<0.0001

UPF Proportion of ultra-processed food intake

[a] Values are means ± standard deviation or numbers (percentages) as appropriate

[b] P-values are based on T-test or chi-square test

[c] Physical activity was classified as low (< 30 minutes of physical activity; equivalent to brisk walking/day), moderate (30-60 min) and high (≥ 60 min)

[d] Body mass index (BMI) was classified as underweight (BMI < 18.5), normal weight (18.5 < BMI < 25), overweight (25 ≤ BMI < 30) and obesity (BM I ≥ 30)

[e] Incident cases of cancer, Type 2 diabetes, hypertension and cardiovascular diseases
