# Supplementary material for: Prospective association between ultra-processed food consumption and incident depressive symptoms in the French NutriNet-Santé cohort
Source: BMC Med. 2019 Apr 15;17:78. doi: 10.1186/s12916-019-1312-y (PMC6463641; doi:10.1186/s12916-019-1312-y)
Supplement: Supplementary file 4 — Figure S1. Dose-response association between ultra-processed food intake and incident depressive symptoms using Restricted Cubic Spline. (PDF 190 kb) [file 12916_2019_1312_MOESM4_ESM.pdf]

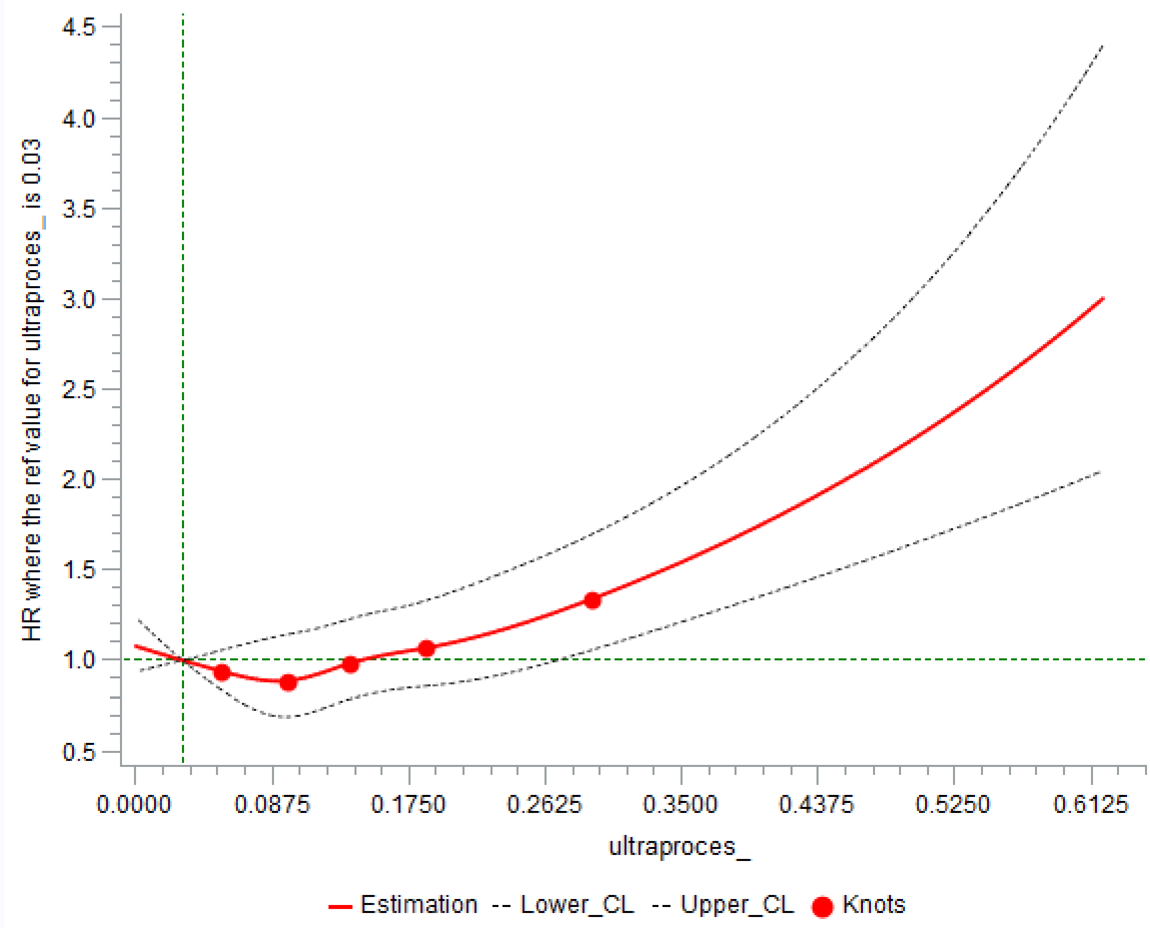

Supplemental Figure 1 Dose-response association between ultra-processed food intake and incident depressive symptoms using Restricted Cubic Spline
