# Supplementary material for: Prospective association between ultra-processed food consumption and incident depressive symptoms in the French NutriNet-Santé cohort
Source: BMC Med. 2019 Apr 15;17:78. doi: 10.1186/s12916-019-1312-y (PMC6463641; doi:10.1186/s12916-019-1312-y)
Supplement: Supplementary file 5 — Table S4. Association between ultra-processed food intake and incident depressive symptoms using other cut-off values to define depressive symptoms, NutriNet-Santé study. (PDF 134 kb) [file 12916_2019_1312_MOESM5_ESM.pdf]

Supplemental Table 4 Association between ultra-processed food intake and incident depressive symptoms using other cut-off values to define depressive symptoms, NutriNet-Santé study [a]

	**Quartile 1**	**Quartile 2**	**Quartile 3**	**Quartile**	**P-trend**	**Continuous [b]**	**P [c]**
				**Cut-off value of 16**			
UPF, range	0%-10%	10%-14%	14%-18%	18%-76%			
UPF, median (IQR)	7% (3%)	12% (2%)	16% (2%)	23% (7%)			
n	5,909	5,910	5,910	5,910		23,639	
Number of cases	827	796	902	1,082		3,607	
Model 1[d]	1 (ref)	0.94 (0.85; 1.04)	1.06 (0.96; 1.17)	1.22 (1.11; 1.34)	<0.0001	1.15 (1.10; 1.20)	<0.0001
Model 2[e]	1 (ref)	0.95 (0.86; 1.05)	1.07 (0.98; 1.18)	1.22 (1.11; 1.35)	<0.0001	1.14 (1.09; 1.19)	<0.0001
Model 3[f]	1 (ref)	0.95 (0.86; 1.05)	1.07 (0.97; 1.19)	1.22 (1.10; 1.35)	<0.0001	1.15 (1.10; 1.20)	<0.0001
Model 4[g]	1 (ref)	0.95 (0.86; 1.05)	1.07 (0.97; 1.18)	1.22 (1.11; 1.35)	<0.0001	1.14 (1.09; 1.19)	<0.0001
Model 5[h]	1 (ref)	0.91 (0.83; 1.01)	1.01 (0.91; 1.11)	1.13 (1.02; 1.24)	0.002	1.10 (1.05; 1.14)	<0.0001
Model 6[i]	1 (ref)	0.93 (0.84; 1.02)	1.05 (0.95; 1.16)	1.21 (1.10; 1.33)	<0.0001	1.14 (1.09; 1.19)	<0.0001
Model 7[j]	1 (ref)	0.91 (0.82; 1.00)	1.00 (0.91; 1.10)	1.13 (1.03; 1.25)	0.002	1.10 (1.05; 1.15)	<0.0001
				**Cut-off value of 19**			
UPF, range	0%-10%	10%-14%	14%-19%	19%-76%			
UPF, median (IQR)	7% (3%)	12% (2%)	16% (2%)	23% (8%)			
n	6,400	6,400	6,400	64,00		25,600	
Number of cases	634	580	697	888		2,799	
Model 1[d]	1 (ref)	0.89 (0.80; 1.00)	1.05 (0.94; 1.17)	1.28 (1.15; 1.42)	<0.0001	1.20 (1.14; 1.25)	<0.0001
Model 2[e]	1 (ref)	0.91 (0.81; 1.02)	1.07 (0.96; 1.19)	1.28 (1.14; 1.42)	<0.0001	1.18 (1.13; 1.23)	<0.0001
Model 3[f]	1 (ref)	0.90 (0.80; 1.01)	1.06 (0.95; 1.19)	1.26 (1.12; 1.41)	<0.0001	1.18 (1.13; 1.24)	<0.0001
Model 4[g]	1 (ref)	0.91 (0.81; 1.02)	1.06 (0.95; 1.19)	1.28 (1.14; 1.42)	<0.0001	1.18 (1.13; 1.23)	<0.0001
Model 5[h]	1 (ref)	0.87 (0.78; 0.98)	1.01 (0.90; 1.13)	1.16 (1.04; 1.30)	0.0004	1.14 (1.08; 1.19)	<0.0001
Model 6[i]	1 (ref)	0.88 (0.79; 0.99)	1.04 (0.93; 1.17)	1.26 (1.13; 1.41)	<0.0001	1.18 (1.13; 1.24)	<0.0001
Model 7[j]	1 (ref)	0.86 (0.77; 0.97)	1.01 (0.90; 1.12)	1.17 (1.05; 1.30)	0.0002	1.14 (1.09; 1.19)	<0.0001

[a] Values are hazard ratios (95% confidence intervals).*CDS* Cognitive Difficulties Scale; *CES-D* Center for Epidemiologic Studies Depression Scale; *IQR* Interquartile Range; *UPF* Proportion of ultra-processed food intake

[b] Hazard ratios for 10% increase in the proportion of ultra-processed food intake

[c] P for continuous variable

[d] Adjusted for age, sex and body mass index

[e] Adjusted for all variables in model 1 + marital status, educational level, occupational categories, household income per consumption unit, residential area, number of 24h-dietary records, inclusion month, energy intake without alcohol, alcohol intake, smoking status and physical activity (main model)

[f] Adjusted for all variables in model 2 + dietary patterns derived from the factor analysis ("Healthy" and "Western" dietary pattern) and intakes of lipids, sodium, and carbohydrates

[g] Adjusted for all variables in model 2 + health events during follow-up (cancer, Type 2 diabetes, hypertension and cardiovascular events)

[h] Adjusted for all variables in model 2 + use of antidepressants during follow-up and baseline CES-D score

[i] Adjusted for all variables in model 2 + CDS score

[j] Adjusted for all variables in model 2 + use of antidepressants during follow-up, baseline CES-D score and CDS score
