# Supplementary material for: Prospective association between ultra-processed food consumption and incident depressive symptoms in the French NutriNet-Santé cohort
Source: BMC Med. 2019 Apr 15;17:78. doi: 10.1186/s12916-019-1312-y (PMC6463641; doi:10.1186/s12916-019-1312-y)
Supplement: Supplementary file 6 — Table S5. Association between ultra-processed food intake (% of energy) and incident depressive symptoms, NutriNet-Santé study. (PDF 125 kb) [file 12916_2019_1312_MOESM6_ESM.pdf]

**Supplemental Table 5** Association between ultra-processed food intake (% of energy) and incident depressive symptoms, NutriNet-Santé study [a]

| | **Quartile 1** | **Quartile 2** | **Quartile 3** | **Quartile 4** | **P-trend** | **Continuous [b]** | **P [c]** |
|---|---|---|---|---|---|---|---|
| UPF_EI, range | 0%-24% | 24%-31% | 31%-39% | 39%-83% | | | |
| UPF_EI, median (IQR) | 19% (6%) | 27% (3%) | 35% (4%) | 46% (9%) | | | |
| n | 6,682 | 6,683 | 6,683 | 6,682 | | 26730 | |
| Number of cases | 444 | 493 | 552 | 732 | | 2,221 | |
| Model 1[d] | 1 (ref) | 1.06 (0.93; 1.21) | 1.11 (0.97; 1.26) | 1.36 (1.19; 1.55) | <0.0001 | 1.12 (1.08; 1.17) | <0.0001 |
| Model 2[e] | 1 (ref) | 1.07 (0.94; 1.21) | 1.11 (0.98; 1.26) | 1.32 (1.15; 1.50) | <0.0001 | 1.10 (1.06; 1.15) | <0.0001 |
| Model 3[f] | 1 (ref) | 1.07 (0.94; 1.22) | 1.12 (0.98; 1.28) | 1.33 (1.15; 1.54) | 0.0001 | 1.11 (1.06; 1.16) | <0.0001 |
| Model 4[g] | 1 (ref) | 1.07 (0.94; 1.22) | 1.12 (0.98; 1.27) | 1.32 (1.16; 1.51) | <0.0001 | 1.10 (1.06; 1.15) | <0.0001 |
| Model 5[h] | 1 (ref) | 1.02 (0.90; 1.16) | 1.07 (0.94; 1.22) | 1.15 (1.01; 1.32) | 0.02 | 1.06 (1.02; 1.10) | 0.004 |
| Model 6[i] | 1 (ref) | 1.03 (0.91; 1.17) | 1.08 (0.95; 1.23) | 1.28 (1.12; 1.46) | 0.0001 | 1.10 (1.06; 1.14) | <0.0001 |
| Model 7[j] | 1 (ref) | 1.00 (0.88; 1.14) | 1.06 (0.93; 1.21) | 1.15 (1.01; 1.31) | 0.02 | 1.06 (1.02; 1.11) | 0.003 |

[a] Values are hazard ratios (95% confidence intervals).*CDS* Cognitive Difficulties Scale; *CES-D* Center for Epidemiologic Studies Depression Scale; *IQR* Interquartile Range; *UPF* Proportion of ultra-processed food intake

[b] Hazard ratios for 10% increase in the proportion of ultra-processed food intake

[c] P for continuous variable

[d] Adjusted for age, sex and body mass index

[e] Adjusted for all variables in model 1 + marital status, educational level, occupational categories, household income per consumption unit, residential area, number of 24h-dietary records, inclusion month, energy intake without alcohol, alcohol intake, smoking status and physical activity (main model)

[f] Adjusted for all variables in model 2 + dietary patterns derived from the factor analysis (“Healthy” and “Western” dietary pattern) and intakes of lipids, sodium, and carbohydrates

[g] Adjusted for all variables in model 2 + health events during follow-up (cancer, Type 2 diabetes, hypertension and cardiovascular events)

[h] Adjusted for all variables in model 2 + use of antidepressants during follow-up and baseline CES-D score

[i] Adjusted for all variables in model 2 + CDS score

[j] Adjusted for all variables in model 2 + use of antidepressants during follow-up, baseline CES-D score and CDS score
