# Supplementary material for: Prospective association between ultra-processed food consumption and incident depressive symptoms in the French NutriNet-Santé cohort
Source: BMC Med. 2019 Apr 15;17:78. doi: 10.1186/s12916-019-1312-y (PMC6463641; doi:10.1186/s12916-019-1312-y)
Supplement: Supplementary file 7 — Table S6. Association between ultra-processed food intake and incident depressive symptoms (considering as cases, only the participants who had depressive symptoms and also reported antidepressant treatment during follow-up), NutriNet-Santé study. (PDF 125 kb) [file 12916_2019_1312_MOESM7_ESM.pdf]

Supplemental Table 6 Association between ultra-processed food intake and incident depressive symptoms (considering as cases, only the participants who had depressive symptoms and also reported antidepressant treatment during follow-up), NutriNet-Santé study [a]

	Quartile 1	Quartile 2	Quartile 3	Quartile 4	P-trend	Continuous [b]	P [c]
UPF_EI, range	0%-24%	24%-31%	31%-39%	39%-83%			
UPF_EI, median (IQR)	19% (6%)	27% (3%)	35% (4%)	46% (9%)			
n	6,682	6,683	6,683	6,682		26,730	
Number of cases	27	22	28	36		113	
Model 1[d]	1 (ref)	0.80 (0.45; 1.41)	1.01 (0.59; 1.72)	1.32 (0.78; 2.21)	0.20	1.44 (1.19; 1.76)	0.0002
Model 2[e]	1 (ref)	0.86 (0.48; 1.51)	1.09 (0.63; 1.87)	1.39 (0.82; 2.37)	0.15	1.43 (1.18; 1.73)	0.0003
Model 3[f]	1 (ref)	0.87 (0.49; 1.54)	1.11 (0.64; 1.94)	1.45 (0.83; 2.54)	0.13	1.51 (1.22; 1.85)	0.0001
Model 4[g]	1 (ref)	0.81 (0.45; 1.43)	1.07 (0.62; 1.84)	1.38 (0.81; 2.35)	0.15	1.43 (1.18; 1.74)	0.0003
Model 5[h]	1 (ref)	0.81 (0.46; 1.42)	1.01 (0.59; 1.74)	1.21 (0.71; 2.07)	0.34	1.36 (1.12; 1.65)	0.002
Model 6[i]	1 (ref)	0.83 (0.47; 1.46)	1.07 (0.62; 1.84)	1.36 (0.80; 2.32)	0.17	1.42 (1.17; 1.72)	0.0004
Model 7[j]	1 (ref)	0.80 (0.45; 1.42)	1.02 (0.59; 1.75)	1.22 (0.71; 2.08)	0.33	1.36 (1.12; 1.65)	0.002

[a] Values are hazard ratios (95% confidence intervals).*CDS* Cognitive Difficulties Scale; *CES-D* Center for Epidemiologic Studies Depression Scale; *IQR* Interquartile Range; *UPF* Proportion of ultra-processed food intake

[b] Hazard ratios for 10% increase in the proportion of ultra-processed food intake

[c] P for continuous variable

[d] Adjusted for age, sex and body mass index

[e] Adjusted for all variables in model 1 + marital status, educational level, occupational categories, household income per consumption unit, residential area, number of 24h-dietary records, inclusion month, energy intake without alcohol, alcohol intake, smoking status and physical activity (main model)

[f] Adjusted for all variables in model 2 + dietary patterns derived from the factor analysis (“Healthy” and “Western” dietary pattern) and intakes of lipids, sodium, and carbohydrates

[g] Adjusted for all variables in model 2 + health events during follow-up (cancer, Type 2 diabetes, hypertension and cardiovascular events)

[h] Adjusted for all variables in model 2 + baseline CES-D score

[i] Adjusted for all variables in model 2 + CDS score

[j] Adjusted for all variables in model 2 + baseline CES-D score and CDS score
